# Occupational Stress and Burnout Among Nurse Managers: Coping Pathways and Leadership Adaptation in Postpandemic Healthcare

**DOI:** 10.1155/jonm/1997548

**Published:** 2026-08-06

**Authors:** Chia-Jung Lin, Wan-Ju Chou, Jia-Huei Wei, Bo Hsiao

**Affiliations:** ^1^ Department of Nursing, College of Health Sciences, Chang Jung Christian University, Tainan, Taiwan, cjcu.edu.tw; ^2^ Department of Psychology, Chung Yuan Christian University, Taoyuan, Taiwan, cycu.edu.tw; ^3^ Department of Nursing, Chi Mei Medical Center, Tainan, Taiwan, chimei.org.tw; ^4^ Department of Information Management, National Yunlin University of Science and Technology, Yunlin, Taiwan, yuntech.edu.tw

**Keywords:** burnout, coping strategies, leadership adaptation, nurse managers, occupational stress, organizational support, workforce well-being

## Abstract

**Background:**

Nurse managers (NMs) play a pivotal role in sustaining care quality, workforce stability, and organizational resilience, yet chronic managerial stress and emotional demands have led to increasing burnout in this group. Understanding the association of coping strategies with this phenomenon is essential to supporting leadership sustainability.

**Aim:**

To examine how problem‐focused coping (PFC) and emotion‐focused coping (EFC) strategies mediate the relationship between occupational stress and burnout among NMs and to identify demographic factors associated with burnout.

**Methods:**

The study adopted a cross‐sectional correlational design and collected data from 116 NMs in two hospitals in southern Taiwan using validated self‐report instruments. Mediation analyses were conducted using bootstrapped regression models (PROCESS Model 4) with 5000 resamples.

**Results:**

Occupational stress correlated positively with burnout (*r* = 0.58, *p* < 0.001). EFC significantly mediated this relationship (*B* = 5.01, 95% CI [2.39, 8.07]), amplifying stress effects and was associated with higher burnout levels, whereas PFC showed no significant mediation. Burnout was significantly associated with younger age, fewer children, and poorer perceived physical condition.

**Conclusions:**

EFC was associated with higher burnout levels under conditions of elevated occupational stress, whereas PFC offered limited protection without organizational reinforcement. These findings refine the stress‐coping framework by demonstrating that coping’s effectiveness depends on contextual and institutional conditions and provide actionable insights for leadership development and workforce sustainability in healthcare organizations.

**Implications for Nursing Management:**

Leadership development programs should incorporate emotional regulation, mentoring, and organizational support systems to strengthen adaptive capacity among NMs. Institutional policies that balance workload and promote psychological well‐being are crucial to sustaining effective nursing leadership.

## 1. Introduction

Nursing management is fundamental to maintaining care quality, workforce stability, and the overall resilience of healthcare organizations. Within the hospital hierarchy, nurse managers (NMs) play a uniquely demanding role that connects clinical practice with administrative oversight. They are expected to supervise staff, allocate resources, and ensure compliance with care standards while maintaining patient safety and team morale. This dual set of responsibilities exposes them to persistent occupational stress and increases their susceptibility to burnout—a syndrome marked by emotional exhaustion, depersonalization, and reduced personal accomplishment.

Around the world, nurse leader burnout has become a pressing concern for both workforce sustainability and healthcare performance, and the problem that became even more visible during the COVID‐19 pandemic [1, 2]. The WHO announced the end of the COVID‐19 public health emergency in May 2023 [3], yet NMs in Taiwan continued to face an increased postpandemic workload, staffing shortages, and accumulated administrative pressures during the data collection period. Accordingly, this study captures a critical postpeak but high‐burden transitional phase. The pandemic magnified long‐standing challenges in nursing leadership, including chronic staff shortages, rising turnover intentions, and constant organizational change. NMs often bore the brunt of these pressures while safeguarding the well‐being of frontline staff. International studies have reported that more than one‐third of NMs experienced moderate to severe burnout during this period [2, 4]. In Taiwan, NMs operate within a healthcare system characterized by long working hours, frequent administrative audits associated with hospital accreditation and quality assurance, and relatively hierarchical organizational structures. Previous studies show that these structural and managerial features place substantial administrative and emotional demands on NMs and may further intensify occupational stress in managerial nursing roles [2, 4]. These conditions underscore the need to examine not only the direct effects of occupational stress but also the psychological pathways through which stress contributes to burnout.

Beyond the immediate psychological burden, these challenges reveal the structural fragility of nursing leadership systems. NMs serve as the critical link between policy directives and frontline implementation, and their ability to maintain composure under stress directly affects staff morale, retention, and patient safety. Understanding how managerial coping operates within this organizational context, therefore, provides not only psychological insight but also the managerial value, as it informs workforce sustainability strategies and leadership development in postpandemic healthcare.

Understanding coping strategies is central to explaining how individuals respond to workplace stress. Lazarus and Folkman’s [5] transactional model of stress and coping describes coping as a dynamic process in which individuals appraise stressors and mobilize cognitive and behavioral resources to restore equilibrium. Within this framework, problem‐focused coping (PFC) denotes efforts such as planning, problem‐solving, and time management aimed at changing the source of stress, whereas emotion‐focused coping (EFC) involves regulating emotional reactions through avoidance, denial, or emotional release. Although PFC is generally adaptive, EFC—while offering temporary relief—may worsen burnout when relied upon under chronic or uncontrollable stress [6, 7].

Despite NMs’ critical leadership role, empirical research on their coping mechanisms remains limited. Most prior studies [8, 9] have focused on staff nurses, leaving a gap in understanding how managerial nurses—who confront distinct role ambiguity, administrative accountability, and emotional strain—experience and manage stress. Furthermore, many studies [10, 11] conceptualize coping as a moderator, assuming it buffers stress effects, rather than as a mediator that explains how stress translates into burnout, yet a mediation perspective aligns more closely with the appraisal–coping–outcome sequence proposed by Lazarus and Folkman and provides richer insight into the psychological process by which stress evolves into burnout.

While Lazarus and Folkman’s transactional model of stress and coping is a general psychological framework, most empirical applications have focused on individual‐level stress responses among clinical staff [5]. This study extends these applications by situating coping processes within the organizational and leadership context of NMs. While the model has been widely tested among clinical nurses, its implications for those in managerial roles remain underexplored. By integrating organizational stressors with individual coping mechanisms, this research provides a contextualized understanding of stress adaptation in managerial nursing practice and contributes to a more nuanced, leadership‐oriented interpretation of the stress–coping process.

The insights drawn from this study are expected to inform evidence‐based interventions that promote managerial resilience, guide leadership training curricula, and help hospital administrators design supportive systems that prevent burnout among NMs. Less is known about how coping processes operate within leadership roles such as NMs, who face distinct administrative accountability and emotional demands. Accordingly, this study posed the following research questions: (1) How are occupational stress and coping strategies associated with burnout among NMs? (2) What demographic or contextual factors are associated with adaptive capacity in managerial roles?

Ultimately, this study contributes to the global discussion of sustaining nursing leadership in the postpandemic era. By elucidating how coping mechanisms are associated with lower or higher levels of burnout under organizational pressure, the study provides both theoretical and practical guidance for developing evidence‐based strategies that support the psychological well‐being and leadership capacity of NMs worldwide.

## 2. Background

Occupational stress has long been recognized as a critical factor affecting nurses’ well‐being and organizational performance. It arises when perceived job demands exceed an individual’s coping capacity, resulting in psychological strain and, in severe cases, burnout [12]. For NMs, occupational stress reflects the cumulative impact of clinical accountability, administrative workload, and interpersonal pressures. Unlike frontline nurses, NMs must simultaneously address top‐down institutional directives and bottom‐up staff concerns, positioning them at the crossroads of organizational expectations and emotional labor [2, 4].

### 2.1. Occupational Stress and Burnout in Nursing Leadership

Burnout—characterized by emotional exhaustion, depersonalization, and a diminished sense of personal accomplishment—is a frequent consequence of prolonged occupational stress [13]. Within nursing management, burnout undermines leadership effectiveness, decision‐making, and ultimately patient safety [14]. Studies consistently identify excessive workload, inadequate staffing, and ambiguous administrative expectations as the strongest predictors of managerial burnout [15, 16]. The COVID‐19 crisis further exacerbated these issues, heightening moral distress and emotional fatigue among those responsible for leading teams through uncertainty [17].

Postpandemic evidence also indicates that burnout among NMs differs in quality and intensity from that experienced by clinical nurses [2]. Understanding burnout in this group, therefore, requires attention to both external stressors and internal coping dynamics.

The five domains of occupational stress assessed in this study—workload, administrative management, role pressure, professional competence, and interpersonal relationships—represent conceptually distinct managerial demands. Workload stress reflects task volume and time constraints; administrative management stress pertains to organizational expectations and accountability; role pressure involves ambiguity and conflicting expectations; professional competence concerns self‐perceived adequacy; and interpersonal relationship stress arises from team dynamics and conflict. Prior research has recognized that these domains may activate different psychological appraisal processes and, consequently, different coping responses [2, 16]. Examining each dimension separately enables a more nuanced understanding of how specific managerial stressors relate to burnout through coping mechanisms.

### 2.2. Coping Strategies: From Individual Response to Systemic Adaptation

Coping encompasses the cognitive and behavioral strategies that individuals employ to handle stressors perceived as threatening or exceeding their resources [5]. Within nursing, two major coping dimensions—PFC and EFC—have been extensively investigated. PFC involves active methods such as planning, prioritizing, or seeking instrumental support, whereas EFC embraces emotion‐oriented actions such as avoidance, withdrawal, or emotional suppression [6].

PFC generally fosters problem resolution and is considered adaptive. EFC, while occasionally useful for short‐term emotional regulation, may become counterproductive when it perpetuates avoidance or detachment. Under conditions of intense and uncontrollable stress, as seen during the COVID‐19 pandemic, EFC often dominates, contributing to higher emotional exhaustion and burnout [7, 18].

Importantly, coping should not be viewed solely as an individual capacity; instead, it represents a context‐dependent process shaped by the organizational environment, leadership culture, and available social support. Research indicates that effective coping depends on both personal psychological flexibility and the institution’s ability to provide structural supports, such as participative leadership, equitable workload distribution, and emotional resources [19, 20].

Consequently, examining coping among NMs requires integrating both personal and organizational dimensions—a perspective seldom addressed in previous studies.

### 2.3. The Mediating Role of Coping Between Stress and Burnout

Although the positive association between occupational stress and burnout is well established, the underlying mechanisms remain less clear. Within Lazarus and Folkman’s transactional model, coping operates as a mediating process through which individuals’ appraisals of stress influence outcomes [5]. However, much of the nursing literature has treated coping as a moderating variable that buffers rather than transmits stress effects. Emerging evidence suggests that mediation models may better capture the dynamic process by which stress appraisals evolve into burnout symptoms [6, 21].

In this conceptualization, PFC and EFC represent distinct pathways through which occupational stress may be translated into burnout. PFC is theorized to facilitate adaptive control processes, whereas EFC may heighten emotional strain. However, empirical findings remain mixed; some studies suggest that prolonged workload stress alters the effectiveness and utilization of PFC, whereas others indicate that EFC may temporarily alleviate anxiety responses [7, 21]. These inconsistencies underscore the need to examine coping’s mediating role within specific organizational contexts, particularly among NMs, whose managerial and emotional demands differ substantially from those of frontline staff.

## 3. Methods

### 3.1. Design

The study adopted a cross‐sectional correlational design to investigate how coping strategies mediate the relationship between occupational stress and burnout among NMs during the COVID‐19 pandemic. The study adhered to the Strengthening the Reporting of Observational Studies in Epidemiology (STROBE) guidelines for cross‐sectional research, ensuring methodological transparency and rigor.

### 3.2. Setting and Participants

Data were collected in April and May 2023 from two hospitals in southern Taiwan—one medical center and one regional hospital—to capture perspectives across both tertiary and secondary healthcare settings. Eligible participants included NMs, such as head nurses and assistant head nurses, who had held their current managerial position for at least 3 months. Individuals in temporary or acting roles and those who submitted incomplete questionnaires were excluded.

Anonymity was ensured to reduce common method bias, and validated instruments were used. Mediation effects were examined using bootstrapping to obtain robust estimates of indirect effects.

A total of 119 questionnaires were distributed, and 116 valid responses were obtained from NMs, representing a 93.3% response rate. To evaluate the adequacy of the sample size for detecting mediation effects, Monte Carlo power simulations were conducted using the method developed by Selig and Preacher [22] (https://quantpsy.org/medmc/medmc.htm). Based on the observed path coefficients (a and b paths) and their associated standard errors from the PROCESS mediation models, 20,000 Monte Carlo replications were generated. The simulation results indicated that the indirect effects were statistically detectable with the existing sample size (*N* = 116), suggesting adequate statistical power for the mediation analyses. This simulation‐based approach is recommended over conventional regression‐based power analysis because it directly accounts for the joint distribution of the a and b paths in mediation models [23, 24].

Table 1 presents the participants’ demographic and occupational characteristics (*N* = 116). Most participants were female (95.70%), but five were male (4.30%). The participants’ mean age was 43.96 years (*SD* = 5.47; range: 31–61). Most participants were aged either 41–45 years (44.0%) or ≥ 46 years (33.60%), whereas 22.40% were aged ≤ 40 years. Nearly two‐thirds held a master’s degree (65.50%), and the majority were married or cohabiting (69.80%).

**TABLE 1:** General characteristics of the NMs (*N* = 116).

Variables	*n*	Percentage (%)
*Gender*		
Female	111	95.70
Male	5	4.30

*Age*		
≤ 40 years	26	22.41
41–45 years	51	43.97
≥ 46 years	39	33.62

*Education*		
Bachelor	40	34.50
Master	76	65.50

*Marital*		
Single^1^	35	30.20
Married/cohabiting	81	69.80

*Number of children*		
None	42	36.21
1	26	22.41
2	48	41.38

*Title*		
Head nurse	62	53.45
Assistant Head Nurse	54	46.55

*Work unit*		
Internal medicine	28	24.14
Surgery	17	14.66
Obstetrics and gynecology	12	10.34
Emergency unit^2^	33	28.45
Special unit^3^	26	22.41

*Clinical experience*		
≤ 15 years	17	14.66
16–20 years	44	37.93
21–25 years	26	22.41
≥ 26 years	29	25.00

*Administrative experience*		
≤ 5 years	36	31.03
6–10 years	34	29.31
≥ 11 years	46	39.66

*Hospital level*		
Medical center	63	54.31
Regional hospital	53	45.69

*Perceived physical condition*		
Poor	6	5.17
Average	61	52.59
Good or above	49	42.24

^1^Includes unmarried (*n* = 34) and divorced (*n* = 1).

^2^Intensive care unit, emergency department, burn unit, and subacute respiratory care unit.

^3^Outpatient, operating room, dialysis room, psychiatry, and palliative care.

More than half the participants served as head nurses (53.45%), and 46.55% were assistant head nurses. Slightly more than half worked in medical centers (54.31%), whereas 45.69% were employed in regional hospitals. Participants were distributed across various clinical units, with the largest proportions working in emergency‐related units (28.45%) and internal medicine units (24.14%), followed by special units (22.41%), surgical units (14.66%), and obstetrics and gynecology units (10.34%). The mean length of clinical experience was 21.69 years (*SD* = 6.20; range: 9–41), and mean administrative experience was 9.66 years (*SD* = 6.42; range: 1–31). Regarding self‐rated physical condition, most participants perceived their health as average (52.59%), followed by good or above (42.24%), but 5.17% reported poor physical condition.

### 3.3. Instruments

#### 3.3.1. Demographic and Occupational Characteristics

An 11‐item demographic questionnaire was developed to collect information on participants’ gender, age, marital status, education level, number of children, years of clinical and administrative experience, work unit, hospital level, and self‐rated physical health.

#### 3.3.2. Occupational Stress Scale

Occupational stress was measured using a 20‐item scale originally developed by Liao [25] and subsequently refined based on recent studies [4, 26]. The instrument assesses five domains: workload (5 items), administrative management (6 items), role stress (3 items), professional competence (3 items), and interpersonal relationships (3 items). Items were rated on a 5‐point Likert scale ranging from 1 (“*strongly disagree*”) to 5 (“*strongly agree*”), with higher scores indicating greater perceived stress. In the present study, the Cronbach’s *α* for the overall scale was 0.94, with subscale reliabilities ranging from 0.71 to 0.83, demonstrating excellent internal consistency.

#### 3.3.3. Work‐Related Burnout Inventory

Burnout was assessed using the Work‐Related Burnout Inventory developed by Yeh et al. [27], which consists of five items rated on a 5‐point Likert scale. Total scores were converted to a 0–100 scale, with higher scores representing greater levels of burnout. This instrument has been widely validated among healthcare professionals in Taiwan. In the current sample, Cronbach’s *α* was 0.88, indicating strong reliability.

#### 3.3.4. Coping Strategy Scale

Coping strategies were measured using the 17‐item Coping Strategy Scale [28], comprising two subscales: PFC (8 items) and EFC (9 items). Responses were recorded on a 5‐point Likert scale, with higher scores reflecting more frequent use of each coping style. In this study, the Cronbach’s *α* values were 0.75 for PFC, 0.77 for EFC, and 0.72 for the total scale, indicating acceptable reliability.

#### 3.3.5. Validity and Reliability

All instruments used in this study have demonstrated established psychometric properties in previous research. Content validity assessment was conducted only to ensure contextual appropriateness for NMs. Content validity was evaluated by a panel of five experts, including two senior nursing administrators and three academic researchers, who reviewed item clarity and relevance. The item‐level content validity index ranged from 0.80 to 1.00 for the Occupational Stress Scale and reached 1.00 for the Coping Strategy Scale, confirming strong content validity. A pilot test with five NMs was conducted to ensure the clarity and appropriateness of the questionnaire items.

### 3.4. Data Collection

Following institutional approval, the research team collaborated with the nursing directors of each participating hospital to facilitate participant recruitment. Questionnaires, accompanied by informed consent forms, were distributed in sealed envelopes and returned anonymously within 2 weeks. Participants required approximately 15–20 min to complete the survey. To ensure confidentiality, no identifying information was collected. All data were stored in password‐protected files accessible only to the research team.

### 3.5. Data Analysis

Based on theoretical relevance and prior empirical evidence, three variables—age, number of children, and perceived physical condition—were included as covariates in all mediation models to control for potential confounding effects. Prior studies have documented associations between age and burnout [17]; self‐rated health and psychological strain [29]; and family structure and coping behavior among healthcare professionals [26]. Preliminary analyses indicated that these variables were significantly associated with burnout or coping strategies (*p* < 0.05). The bootstrapping approach was selected because it does not rely on normality assumptions and provides more robust confidence interval estimation for mediation models. Data were analyzed using IBM SPSS Statistics Version 23.0. Descriptive statistics, including means, standard deviations, and frequency distributions, were calculated to summarize participant characteristics and main study variables. Pearson correlation coefficients were used to examine the relationships among occupational stress, coping strategies, and burnout.

As discussed in Section 2.1, each stress dimension was modeled separately to preserve the interpretability of dimension‐specific indirect effects and to avoid multicollinearity under the existing sample size. To control for Type I error inflation due to multiple testing across five dimension‐specific models, a Bonferroni‐adjusted significance threshold (*α* = 0.05/5 = 0.01) was applied when evaluating the dimension‐specific indirect effects. To test the mediating effects of PFC and EFC, bootstrapped regression analyses were conducted using the PROCESS macro (Model 4; [30]). A total of 5000 bootstrap samples were drawn to generate bias‐corrected 95% confidence intervals. Mediation was considered significant when the confidence interval for the indirect effect did not include zero. Statistical significance was set at *p* < 0.05 for all analyses. This analytic approach enabled a robust estimation of both direct and indirect relationships, reducing the bias associated with nonnormal sampling distributions in mediation testing. All statistical assumptions were checked, and multicollinearity was within acceptable limits variance inflation factor (VIF) < 2.5).

To assess the potential influence of common method bias, Harman’s single‐factor test was conducted by entering all measurement items into an unrotated principal component analysis [31]. The results show that the first factor accounted for 27.84% of the total variance, which is well below the commonly suggested threshold of 50%. This result suggests that a single common factor does not account for the majority of variance in the data, which is consistent with the interpretation that severe common method bias is unlikely in the present study. However, it should be noted that Harman’s single‐factor test is an imperfect and conservative indicator; it can detect only the most extreme cases of common method variance and cannot fully exclude the possibility that common method bias influenced some associations. Accordingly, the findings should be interpreted with this limitation in mind.

### 3.6. Ethical Considerations

Ethical approval for this study was obtained from the Institutional Review Boards of both participating hospitals (IRB nos. 11,203‐013 and TMANH112‐REC008). All participants were informed about the study’s purpose, procedures, voluntary nature, and confidentiality safeguards. Participants provided written informed consent prior to answering the questionnaire.

To protect anonymity, no identifying information was collected, and all completed questionnaires were coded numerically. Data were stored in encrypted, password‐protected files accessible only to the research team. The study complied fully with ethical standards for research involving human participants and followed the principles outlined in the Declaration of Helsinki.

## 4. Results

### 4.1. Descriptive Statistics of Key Variables

Table 2 summarizes the descriptive statistics for occupational stress, burnout, and coping strategies among NMs. The overall level of occupational stress was moderate (*M* = 3.31 and *SD* = 0.60), with scores ranging from 1.20 to 4.50. Among the five stress dimensions, workload had the highest mean score (*M* = 3.64 and *SD* = 0.57), followed by administrative management (*M* = 3.25 and *SD* = 0.63), interpersonal relationships (*M* = 3.25 and *SD* = 0.78), and role stress (*M* = 3.24 and *SD* = 0.76). Professional competence yielded the lowest mean score (*M* = 3.01 and *SD* = 0.84). Burnout scores showed considerable variability (*M* = 45.26 and *SD* = 18.27; range: 5–100), indicating differing levels of emotional exhaustion and strain across participants. Regarding coping strategies, the overall coping score was 3.15 (*SD* = 0.33 and range: 2.35–4.29). Participants reported higher use of PFC (*M* = 3.83 and *SD* = 0.38) than of EFC (*M* = 2.54 and *SD* = 0.51). The descriptive findings indicate that NMs tended to rely more on PFC strategies than on EFC strategies.

**TABLE 2:** Descriptive statistics of occupational stress dimensions, burnout, and coping strategies (*N* = 116).

Variables	*M*	*SD*	Min	Max
Overall occupational stress	3.31	0.60	1.20	4.50
Workload	3.64	0.57	1.60	4.80
Administrative management	3.25	0.63	1.00	4.67
Role stress	3.24	0.76	1.00	4.67
Professional competence	3.01	0.84	1.00	5.00
Interpersonal relationships	3.25	0.78	1.33	5.00
Burnout	45.26	18.27	5.00	100
Coping strategy	3.15	0.33	2.35	4.29
PFC	3.83	0.38	3.00	5.00
EFC	2.54	0.51	1.44	4.00

*Note*: Min = minimum, Max = maximum.

Abbreviations: EFC = emotion‐focused coping, PFC = problem‐focused coping.

### 4.2. Differences by Demographic Characteristics

Table 3 presents the differences in occupational stress, coping strategies, and burnout across demographic and occupational characteristics. Significant group differences were identified for age, number of children, and perceived physical condition.

**TABLE 3:** Differences in stress, coping, and burnout by demographic characteristics (*N* = 116).

Variables	Occupational stress			Burnout			PFC			EFC			Post hoc
	M	SD	t/F	M	SD	t/F	M	SD	t/F	M	SD	t/F	
Gender			0.914^*t*^			1.287^*t*^			0.481^*t*^			1.424^*t*^	
Female	3.32	0.60		45.72	18.34		3.83	0.38		2.55	0.51		
Male	3.07	0.74		35.00	14.58		3.75	0.48		2.22	0.38		
Age			1.738^*F*^			4.239^∗^ ^*F*^			0.203^*F*^			2.048^*F*^	Burnout: A > B (*p* = 0.050) A > C (*p* = 0.019)
^A^≤ 40 years	3.49	0.51		54.04	20.83		3.79	0.44		2.71	0.59		
^B^41–45 years	3.29	0.60		43.63	18.82		3.84	0.37		2.50	0.56		
^C^ ≥ 46 years	3.22	0.64		41.54	13.72		3.84	0.37		2.47	0.35		
Education			1.801^*t*^			1.721^*t*^			−1.532^*t*^			0.646^*t*^	
Bachelor	3.45	0.50		49.25	21.59		3.76	0.40		2.58	0.59		
Master	3.24	0.64		43.16	16.02		3.87	0.37		2.52	0.46		
Marital			1.330^*t*^			1.400^*t*^			0.222^*t*^			1.754^*t*^	
Single^1^	3.42	0.59		48.86	19.48		3.84	0.44		2.66	0.53		
Married/cohabiting	3.26	0.60		43.70	17.62		3.83	0.36		2.48	0.50		
Number of children			1.624^*F*^			1.751^*F*^			0.512^*F*^			3.038^∗^ ^*F*^	EFC: A > C (*p* = 0.010)
^A^None	3.43	0.59		50.12	20.08		3.79	0.43		2.70	0.51		
^B^1	3.11	0.65		41.15	20.99		3.89	0.31		2.56	0.56		
^C^2	3.31	0.57		43.23	14.01		3.83	0.38		2.38	0.44		
Title			−0.182^*t*^			−0.366^*t*^			0.116^*t*^			0.461^*t*^	
Head nurse	3.30	0.61		44.68	16.39		3.83	0.39		2.56	0.44		
Assistant Head Nurse	3.32	0.59		45.93	20.35		3.83	0.38		2.51	0.58		
Work unit			0.192^*F*^			1.289^*F*^			1.713^*F*^			0.509^*F*^	
Internal medicine	3.24	0.64		43.21	20.01		3.87	0.33		2.52	0.61		
Surgery	3.28	0.62		41.18	16.54		3.81	0.37		2.47	0.51		
Obstetrics and gynecology	3.32	0.56		46.67	15.57		3.64	0.40		2.56	0.39		
Emergency unit	3.35	0.61		50.91	20.21		3.94	0.35		2.64	0.51		
Special unit	3.36	0.59		42.31	15.18		3.76	0.44		2.47	0.45		
Clinical experience			0.979^*F*^			2.680^*F*^			0.861^*F*^			0.696^*F*^	
≤ 15 years	3.49	0.56		51.18	20.66		3.76	0.48		2.63	0.60		
16–20 years	3.31	0.52		43.75	17.19		3.82	0.36		2.55	0.56		
21–25 years	3.34	0.62		50.77	21.29		3.93	0.40		2.58	0.52		
≥ 26 years	3.18	0.71		39.14	13.30		3.80	0.35		2.43	0.36		
Administrative experience			1.008^*F*^			0.383^*F*^			2.989^*F*^			1.771^*F*^	
≤ 5 years	3.42	0.60		47.36	22.34		3.88	0.42		2.55	0.65		
6–10 years	3.23	0.58		45.00	14.87		3.70	0.34		2.66	0.41		
≥ 11 years	3.28	0.62		43.80	17.23		3.89	0.36		2.44	0.44		
Hospital level			1.008^*t*^			0.495^*t*^			1.238^*t*^			1.336^*t*^	
Medical center	3.36	0.61		46.03	17.99		3.87	0.34		2.60	0.52		
Regional hospital	3.25	0.59		44.34	18.73		3.78	0.43		2.47	0.49		
Perceived physical condition			2.599^*F*^			4.280^∗^ ^*F*^			0.708^*F*^			0.883^*F*^	Burnout: A > C (*p* = 0.049)
^A^Poor	3.60	0.57		59.17	17.72		3.81	0.53		2.57	0.41		
^B^Average	3.39	0.51		47.79	18.72		3.79	0.41		2.59	0.52		
^C^Good or above	3.17	0.68		40.41	16.52		3.88	0.33		2.46	0.50		

*Note: M* = mean, post hoc comparisons were conducted using the Bonferroni correction; *t* = independent *t*‐test; *F* = Analysis of variance.

Abbreviations: EFC = emotion‐focused coping, PFC = problem‐focused coping, *SD* = standard deviation.

^∗^ *p* < 0.05.

Age was significantly associated with burnout (*F* = 4.239, *p* < 0.05). Post hoc comparisons using the Bonferroni correction indicate that NMs aged ≤ 40 years reported significantly higher burnout scores than those aged 41–45 years (*p* = 0.050) or ≥ 46 years (*p* = 0.019). No significant age differences were observed for occupational stress or coping strategies. Number of children was significantly associated with EFC (*F* = 3.038, *p* < 0.05). Participants without children reported higher EFC scores than those with two children (*p* = 0.010). No significant differences were found for occupational stress, burnout, or PFC. Perceived physical condition was significantly related to burnout (*F* = 4.280, *p* < 0.05). Participants who rated their health as poor reported significantly higher burnout levels than those who rated their health as good or above (*p* = 0.049). No significant differences were observed in occupational stress, burnout, or coping strategies with respect to gender, education level, marital status, job title, work unit, clinical experience, administrative experience, or hospital level.

### 4.3. Correlations Among Stress, Coping, and Burnout

Table 4 presents the Pearson correlation coefficients among age, years of administrative service, occupational stress, coping strategies, and burnout. Occupational stress was strongly and positively correlated with burnout (*r* = 0.581, *p* < 0.001). All five dimensions of occupational stress—workload, administrative management, role pressure, professional competence, and interpersonal relationships—were also significantly correlated with burnout (*r* = 0.446–0.592, all *p* < 0.001).

**TABLE 4:** Correlations among age, tenure, stress, coping, and burnout (*N* = 116).

Variable	Age	Y	OS	x1	x2	x3	x4	x5	PFC	EFC	B
Age											
Years of administrative service (Y)	0.665^∗∗∗^										
Occupational stress (OS)	−0.152	−0.133									
Workload (x1)	−0.145	−0.065	0.856^∗∗∗^								
Administration (x2)	−0.080	−0.038	0.915^∗∗∗^	0.727^∗∗∗^							
Role pressure (x3)	−0.201^∗^	−0.177	0.887^∗∗∗^	0.738^∗∗∗^	0.758^∗∗∗^						
Professional knowledge (x4)	−0.114	−0.173	0.823^∗∗∗^	0.583^∗∗∗^	0.673^∗∗∗^	0.670^∗∗∗^					
Interpersonal relationship (x5)	−0.156	−0.183^∗^	0.871^∗∗∗^	0.664^∗∗∗^	0.736^∗∗∗^	0.741^∗∗∗^	0.703^∗∗∗^				
PFC	0.012	−0.018	0.053	0.115	0.064	0.031	0.020	−0.020			
EFC	−0.186^∗^	−0.141	0.437^∗∗∗^	0.432^∗∗∗^	0.332^∗∗∗^	0.393^∗∗∗^	0.349^∗∗∗^	0.428^∗∗∗^	0.024		
Burnout (B)	−0.227^∗^	−0.170	0.581^∗∗∗^	0.592^∗∗∗^	0.446^∗∗∗^	0.503^∗∗∗^	0.514^∗∗∗^	0.505^∗∗∗^	0.101	0.595^∗∗∗^	

Abbreviations: EFC = emotion‐focused coping, PFC = problem‐focused coping.

^∗^ *p* < .05.

^∗∗^ *p* < .01.

^∗∗∗^ *p* < .001.

EFC showed a moderate positive correlation with occupational stress (*r* = 0.437, *p* < 0.001) and burnout (*r* = 0.595, *p* < 0.001). In contrast, PFC was not significantly correlated with occupational stress (*r* = 0.053, *p* > 0.05) or burnout (*r* = 0.101, *p* > 0.05).

Age was negatively correlated with burnout (*r* = −0.227, *p* < 0.05) and with EFC (*r* = −0.186, *p* < 0.05). However, years of administrative service were not significantly associated with burnout (*r* = −0.170, *p* > 0.05). Age was positively correlated with years of administrative service (*r* = 0.665, *p* < 0.001), indicating a substantial association between these two variables.

### 4.4. Mediation Analyses

Bootstrapped mediation analyses were conducted using the PROCESS macro (Model 4; [30]) to examine whether coping strategies mediated the association between occupational stress and burnout. Age, number of children, and perceived physical condition were included as covariates in all mediation models to control for potential confounding effects.

As shown in Table 5, occupational stress demonstrated a significant total effect on burnout (*B* = 15.984, 95% CI [11.338, 20.630], and *p* < 0.001). After accounting for coping strategies and covariates, the direct effect of occupational stress on burnout remained significant (*B* = 10.730, 95% CI [6.161, 15.299], and *p* < 0.001). Regarding indirect effects, EFC significantly mediated the relationship between occupational stress and burnout (*B* = 5.014, 95% CI [2.389, 8.073], and *p* < 0.001), whereas PFC did not demonstrate a significant mediating effect (*B* = 0.240 and 95% CI [−0.428, 1.083]).

**TABLE 5:** Total, direct, and indirect effects of occupational stress on burnout.

	**Process**						**Monte Carlo 95% CI**	
**Predictor**	**B**	**β**	**SE**	**95% CILL**	**95% CIUL**	**p**	**LLCI**	**ULCI**

*Direct effect*								
Model A (occupational stress⟶burnout)	10.730	0.353	2.306	6.161	15.299	< 0.001	—	—
Model B (workload⟶burnout)	11.666	0.361	2.499	6.711	16.621	< 0.001	—	—
Model C (administration⟶burnout)	7.421	0.257	2.125	3.210	11.632	< 0.01	—	—
Model D (role Pressure⟶burnout)	6.324	0.263	1.934	2.490	10.157	< 0.001	—	—
Model E (professional⟶burnout)	6.775	0.311	1.612	3.580	9.970	< 0.01	—	—
Model F (interpersonal⟶burnout)	6.945	0.295	1.796	3.387	10.504	< 0.01	—	—

*Indirect effect (PFC)*								
Model A (occupational stress⟶PFC⟶burnout)	0.240	—	0.365	−0.428	1.083	—	−0.3764	1.171
Model B (workload⟶PFC ⟶burnout)	0.351	—	0.480	−0.425	1.527	—	−0.4025	1.474
Model C (administration⟶PFC⟶burnout)	0.224	—	0.350	−0.521	0.946	—	−0.3681	1.108
Model D (role Pressure⟶PFC⟶burnout)	0.181	—	0.286	−0.259	0.878	—	−0.3603	0.9667
Model E (professional⟶PFC⟶burnout)	0.105	—	0.273	−0.537	0.629	—	−0.4044	0.7513
Model F (interpersonal⟶PFC⟶burnout)	−0.016	—	0.297	−0.704	0.576	—	−0.6885	0.6414

*Indirect effect (EFC)—*								
Model A (occupational stress⟶EFC⟶burnout)	5.014	—	1.475	2.389	8.073	< 0.001	2.529	8.078
Model B (workload⟶EFC ⟶burnout)	5.300	—	1.670	2.521	9.021	< 0.001	2.591	8.605
Model C (administration⟶EFC⟶burnout)	4.060	—	1.283	1.837	6.832	< 0.001	1.628	6.988
Model D (role pressure⟶EFC⟶burnout)	3.195	—	1.001	1.295	5.231	< 0.001	2.075	6.864
Model E (professional⟶EFC⟶burnout)	3.587	—	1.079	1.562	5.804	< 0.001	1.39	5.363
Model F (interpersonal⟶EFC⟶burnout)	3.966	—	1.127	1.908	6.368	< 0.001	1.988	6.4

*Total effect*								
Model A (occupational stress⟶burnout)	15.984	0.526	2.344	11.338	20.630	< 0.001	—	—
Model B (workload⟶burnout)	17.317	0.536	2.541	12.281	22.353	< 0.001	—	—
Model C (administration⟶burnout)	11.705	0.406	2.335	7.078	16.333	< 0.001	—	—
Model D (role Pressure⟶burnout)	10.756	0.448	2.033	6.729	14.784	< 0.001	—	—
Model E (professional⟶burnout)	10.074	0.462	1.749	6.609	13.539	< 0.001	—	—
Model F (interpersonal⟶burnout)	10.895	0.463	1.864	7.202	14.588	< 0.001	—	—

*Note*: *B* = unstandardized coefficients; *β* ***= *** standardized coefficients.

Abbreviations: EFC = emotion‐focused coping, EFC = emotion‐focused coping, PFC = problem‐focused coping, ULCI = upper limit confidence interval.

Further analyses examining each dimension of occupational stress indicated that EFC consistently mediated the associations between burnout and workload (*B* = 5.300 and 95% CI [2.521, 9.021]), administrative management (*B* = 4.060 and 95% CI [1.837, 6.832]), role pressure (*B* = 3.195 and 95% CI [1.295, 5.231]), professional competence (*B* = 3.587 and 95% CI [1.562, 5.804]), and interpersonal relationships (*B* = 3.966 and 95% CI [1.908, 6.368]). In contrast, PFC did not demonstrate significant indirect effects in any of the models.

To further examine the robustness of the mediation effects, Monte Carlo simulations were conducted based on the observed path coefficients and variance estimates. The Monte Carlo 95% confidence interval for the indirect effect in the overall occupational stress model ranged from 2.529 to 8.078, which did not include zero, confirming a statistically significant mediation effect. Similar patterns were observed across all occupational stress dimensions.

**FIGURE 1:**
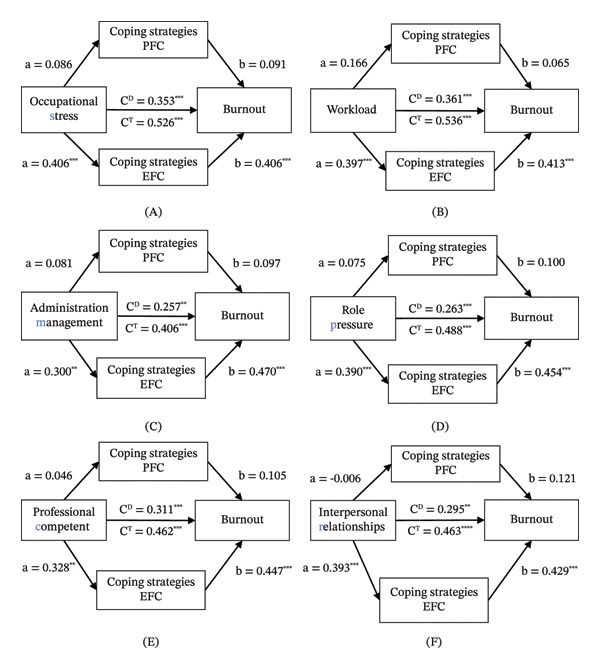
Conceptual model of occupational stress and burnout. (A) Occupational stress. (B) Workload stress. (C) Administration management stress. (D) Role pressure stress. (E) Professional competence stress. (F) Interpersonal relationships stress. Note: *β* = standardized path coefficients are presented in Figure 1; PFC = problem‐focused coping; EFC = emotion‐focused coping.

Figure 1 illustrates the mediation pathways identified in the analyses. In the overall model, occupational stress was positively associated with EFC (*a* = 0.406, *p* < 0.001), and EFC was positively associated with burnout (*b* = 0.406, *p* < 0.001). In contrast, the paths involving PFC were not statistically significant (*a* = 0.086 and *b* = 0.091). The direct effect of occupational stress on burnout remained significant (*β* = 0.353, *p* < 0.001), and the total effect was *β* = 0.526 (*p* < 0.001).

Similar patterns were observed across the five occupational stress dimensions. For workload stress, EFC significantly mediated the relationship with burnout (*a* = 0.397 and *b* = 0.413, both *p* < 0.001), whereas the PFC pathways were not significant (*a* = 0.166 and *b* = 0.065). For administrative management stress, EFC also showed significant mediation (*a* = 0.300, *p* < 0.01; *b* = 0.470, *p* < 0.001), whereas the PFC pathway remained nonsignificant (*a* = 0.081 and *b* = 0.097). For role pressure stress, EFC was significantly associated with burnout (*a* = 0.390 and *b* = 0.454, both *p* < 0.001), whereas the PFC paths were not significant (*a* = 0.075 and *b* = 0.100). For professional competence stress, EFC remained significant (*a* = 0.328, *p* < 0.01; *b* = 0.447, *p* < 0.001), whereas the PFC paths were small and nonsignificant (*a* = 0.046 and *b* = 0.105). For interpersonal relationship stress, EFC again demonstrated significant associations (*a* = 0.393 and *b* = 0.429, both *p* < 0.001), whereas PFC showed no significant pathway (*a* = −0.006 and *b* = 0.121).

### 4.5. Sensitivity Analysis

A sensitivity analysis was conducted using the female‐only subsample (*n* = 111) to evaluate whether the inclusion of male participants affected the findings. The results were highly consistent with those obtained from the full sample (*N* = 116), with no meaningful changes in the direction or statistical significance of the observed relationships.

### 4.6. Summary of Findings

Demographic and contextual factors, including age, health status, family context, and hospital level, were incorporated as covariates and contributed to explaining individual variability in burnout without altering the overall pattern of mediation effects.

Figure 1 presents the validated conceptual model. The findings confirm that occupational stress was strongly associated with burnout. EFC emerged as a significant mediating pathway through which occupational stress was associated with higher burnout levels. In contrast, though PFC, was more frequently employed, it did not significantly mediate the relationship between occupational stress and burnout. Overall, the results indicate the following:

• Occupational stress was a key determinant of burnout among NMs, particularly stressors related to workload and administrative complexity,
• EFC was associated with higher levels of burnout under conditions of elevated stress, and
• PFC, when considered in isolation, appeared to offer limited protection against burnout, underscoring the importance of organizational‐level support.

Collectively, these findings extend the transactional model of stress and coping by demonstrating that coping effectiveness depends not only on individual strategies but also on the organizational conditions that shape adaptive responses.

Figure 1 illustrates the conceptual model that guided this study, grounded in Lazarus and Folkman’s [5] transactional model of stress and coping. Within this framework, occupational stress represents the external and organizational demands perceived by NMs, including workload, administrative expectations, and interpersonal pressures. These stressors are associated with burnout, conceptualized as a multidimensional syndrome encompassing emotional exhaustion, depersonalization, and reduced personal accomplishment.

Two major coping pathways are proposed as mediating mechanisms: PFC and EFC. PFC denotes active, task‐oriented efforts to alter or manage the source of stress through planning, time management, or problem‐solving. In contrast, EFC encompasses strategies aimed at regulating emotional responses—such as avoidance, denial, or emotional release—which may offer temporary relief but can exacerbate burnout when overused under chronic stress.

The model incorporates demographic and contextual covariates—age, number of children, perceived physical condition, and hospital level—which were controlled for in the mediation analyses based on their observed associations with burnout and coping (see Table 3)—as adaptive capacity–related factors that may be associated with both coping and burnout outcomes. Through this framework, the study aims to clarify how coping functions as a psychological bridge linking occupational stress to burnout, providing both theoretical insight and managerial guidance for strengthening nurse leader adaptive capacity.

## 5. Discussion and Implications

This study examined the mediating role of coping strategies in the relationship between occupational stress and burnout among NMs during the COVID‐19 pandemic. Three central findings emerged. First, occupational stress demonstrated a strong and positive association with burnout. Second, EFC significantly mediated this relationship and was associated with higher burnout under conditions of chronic stress. Third, PFC, although more frequently used, did not emerge as a significant mediator between organizational pressure and burnout. Moreover, younger age, poorer self‐rated health, and the absence of family support was associated with higher burnout levels, suggesting that both personal and contextual factors shape adaptive capacity among nurse leaders.

Each occupational stress dimension reflects a distinct managerial demand (e.g., workload vs. interpersonal strain) that may trigger different coping processes. To preserve interpretability under a limited sample size, separate mediation models were tested for each stress domain. This approach aligns with prior nursing stress research adopting domain‐specific analytical strategies.

### 5.1. Theoretical Contributions

This study advances the nursing leadership literature by extending the empirical application of Lazarus and Folkman’s [5] transactional model of stress and coping to the context of NMs. While the transactional model conceptualizes stress and coping as a dynamic psychological process involving appraisal and coping at the individual level, prior empirical applications in nursing have largely focused on frontline or nonmanagerial staff. Consequently, limited attention has been paid to how this stress‐coping process operates within leadership roles characterized by managerial accountability, organizational coordination, and emotional labor.

The present findings provide empirical evidence that coping functions as a process mechanism linking occupational stress to burnout among NMs. Our mediation analyses demonstrated that multiple dimensions of occupational stress—including workload demands, administrative management pressures, and interpersonal stress—were indirectly associated with burnout through coping strategies. These results support the core propositions of the transactional model by illustrating how stress appraisal and coping processes unfold in leadership contexts, where stressors are embedded within organizational structures rather than arising solely from individual task demands.

Importantly, this study highlights the contextualized nature of coping effectiveness in nursing leadership roles. The observed mediated pathways indicate that coping strategies did not operate as isolated individual efforts but rather as responses shaped by organizational and managerial conditions. Stressors such as administrative accountability, staffing responsibilities, and interpersonal coordination reflect institutional expectations and structural constraints that influence how coping strategies are deployed and how coping strategies are associated with different levels of burnout. As such, coping effectiveness among NMs depends not only on individual coping approaches but also on the organizational context in which coping occurs.

By demonstrating that the stress–coping–burnout process is contingent on contextual and organizational conditions, this study supports emerging perspectives that emphasize the limitations of individual‐focused coping frameworks in leadership settings. Rather than suggesting any shortcoming of the original transactional model, our findings underscore its adaptability when applied to role‐specific and organizationally embedded stressors. Situating the transactional stress process within nursing leadership offers a more nuanced theoretical understanding of burnout mechanisms among NMs and provides a foundation for integrating transactional stress theory with organizational and leadership perspectives in future research.

Beyond the stress‐coping framework, these findings can be understood through the lens of organizational and leadership theory. NMs often operate with limited decision autonomy despite high accountability, a combination that restricts the effectiveness of PFC and may push individuals toward emotionally oriented responses. Furthermore, the hierarchical structures characteristic of Taiwanese hospital systems may constrain participatory leadership and limit access to peer‐based coping resources [10, 19]. Future research should integrate organizational variables such as perceived organizational support, decision latitude, and leadership style into multilevel models to better understand how structural factors are associated with NMs’ well‐being.

### 5.2. Implications for Nursing Practice

Burnout among NMs poses not only an individual health concern but also a systemic threat to organizational performance, staff morale, and patient safety. The present findings have several practical implications for leadership development and institutional policy.

First, interventions should incorporate structured emotional regulation and mindfulness training within leadership development programs. Because EFC was associated with higher burnout levels under conditions of chronic stress, equipping NMs with techniques for cognitive reframing and emotional self‐regulation may help reduce reliance on maladaptive coping patterns.

Second, organizations should prioritize peer consultation and mentoring systems that promote open dialogue and collective problem‐solving. These support structures can counter the isolation often experienced in managerial roles and provide safe spaces for stress appraisal and reflection.

Third, tailored wellness initiatives are warranted for younger NMs and those reporting poorer health, as these groups are particularly vulnerable. Health promotion efforts—such as flexible scheduling, physical well‐being programs, and counseling services—may enhance retention and sustain leadership capacity.

Fourth, institutional leaders should move beyond the notion of coping as an individual responsibility and adopt a systems‐based resilience framework. Embedding adaptive capacity indicators into managerial evaluation and professional development systems can encourage organizations to view well‐being as a shared responsibility rather than an individual burden.

Lastly, policymakers and hospital administrators should recognize managerial well‐being as a key component of healthcare quality. Investments in workload management, digital workflow optimization, and participatory decision‐making processes can alleviate structural sources of stress and foster a healthier organizational climate.

### 5.3. Limitations and Future Research

Several limitations should be considered when interpreting the findings. First, because this study employed a cross‐sectional design, the results should be interpreted as associations rather than causal relationships. Future longitudinal studies are needed to clarify the temporal relationships among occupational stress, coping strategies, and burnout. Second, the use of self‐reported measures may introduce potential bias related to social desirability or recall concerns. Although Harman’s single‐factor test did not find evidence of a predominant common method factor, as one factor accounted for 27.84% of total variance—well below the 50% threshold— that procedure is recognized as an insensitive test that may fail to detect more subtle method effects [31]. Future studies should consider marker‐variable techniques or procedural remedies such as temporal separation of measurements to more rigorously address this limitation. Third, each occupational stress dimension was examined in separate mediation models, which may increase the risk of inflated Type I error. Although a Bonferroni correction was applied, the findings should still be interpreted with caution. Fourth, the sample was drawn from two hospitals in southern Taiwan and included a small number of male participants, which may limit the generalizability of the findings. Sensitivity analyses excluding male participants yielded results highly consistent with those of the full sample, however, suggesting that the inclusion of male participants did not materially influence the study findings. Future research with larger and more diverse samples could employ structural equation modeling to simultaneously examine multiple stress dimensions and their interrelationships and incorporate organizational variables such as leadership style, perceived support, or decision autonomy to better understand factors associated with NMs’ well‐being.

## 6. Conclusions

The present findings suggest that while NMs employ both PFC and EFC strategies, only the latter was significantly associated with the relationship between occupational stress and burnout and in a direction consistent with heightened burnout risk. These findings suggest that coping strategies in isolation may offer limited protection against burnout when systemic pressures are substantial. Sustained well‐being may be better supported by a multilevel adaptive capacity approach that combines emotional regulation at the individual level with organizational and policy‐level support.

By providing evidence consistent with a psychological pathway connecting stress and burnout, this research contributes to the theoretical refinement of the stress‐coping model and provides practical guidance for developing evidence‐based interventions. Supporting the emotional and structural needs of NMs is not merely an individual or managerial concern—it is fundamental to preserving the stability, quality, and humanity of nursing leadership in postpandemic healthcare systems. These findings provide actionable insights for nursing leadership and workforce sustainability.

### 6.1. Implications for Nursing Management

The study highlights that managerial well‐being should be recognized as a strategic priority within healthcare organizations. EFC, when unaddressed, is associated with higher burnout levels and undermines leadership performance. NMs require both personal coping resources and system‐level support to maintain resilience. Hospitals should integrate emotional regulation training, peer mentoring, and participatory leadership structures into managerial development programs. Additionally, fostering an open culture of stress communication can prevent burnout escalation and sustain high‐performing leadership teams. Embedding well‐being metrics into managerial evaluation frameworks can ensure sustainable leadership capacity.

## Author Contributions

Conceptualization: Chia‐Jung Lin; methodology: Chia‐Jung Lin and Bo Hsiao; formal analysis: Chia‐Jung Lin, Wan‐Ju Chou, and Bo Hsiao; data curation: Chia‐Jung Lin and Jia‐Huei Wei; investigation: Jia‐Huei Wei; funding acquisition: Jia‐Huei Wei; project administration: Chia‐Jung Lin; supervision: Chia‐Jung Lin; validation: Chia‐Jung Lin, Wan‐Ju Chou, and Bo Hsiao; visualization: Chia‐Jung Lin; writing–original draft: Chia‐Jung Lin and Bo Hsiao; writing–review and editing: Chia‐Jung Lin, Wan‐Ju Chou, and Bo Hsiao.

## Funding

This research received no specific grant from any funding agency in the public, commercial, or not‐for‐profit sectors.

## Ethics Statement

This study was approved by the Institutional Review Boards of both participating hospitals (IRB nos. 11,203‐013 and TMANH112‐REC008). All participants were informed of the study’s purpose, procedures, and confidentiality assurances. Written informed consent was obtained prior to participation.

## Conflicts of Interest

The authors declare no conflicts of interest.

## Data Availability

The authors confirm that all data supporting the findings of this study are available from the corresponding author upon reasonable request. The analytical methods were appropriately applied within the research design and context, and all statistical results were interpreted accurately.
